# Antibiotics for gastroenteritis in general practice and out-of-hour services in Norway 2006–15

**DOI:** 10.1093/fampra/cmab080

**Published:** 2021-07-15

**Authors:** Knut Erik Emberland, Knut-Arne Wensaas, Sverre Litleskare, Leo Larsen, Kristine Morch, Sabine Ruths, Guri Rortveit

**Affiliations:** 1 Department of Global Public Health and Primary Care, University of Bergen, Bergen, Norway; 2 Research Unit for General Practice, NORCE Norwegian Research Centre, Bergen, Norway; 3 Norwegian National Advisory Unit on Tropical Infectious Diseases, Department of Medicine, Haukeland University Hospital, Bergen, Norway; 4 Department of Clinical Science, University of Bergen, Bergen, Norway

**Keywords:** Antibiotics, consultation, gastroenterology, health services research, infectious diseases, primary care

## Abstract

**Background:**

When patients with gastroenteritis (GE) seek health care, they are generally managed in primary care. Little is known about the use of antibiotic treatment in these cases.

**Objective:**

The aim of this study was to investigate time trends and patient characteristics associated with antibiotic treatment for GE in Norwegian primary care in a 10-year period.

**Methods:**

We linked data from two nationwide registries, reimbursement claims data from Norwegian primary care (the KUHR database) and The Norwegian Prescription Database, for the period 2006–15. GE consultations were extracted, and courses of systemic antibiotics dispensed within 1 day were included for further analyses.

**Results:**

Antibiotic treatment was linked to 1.8% (*n* = 23 663) of the 1 279 867 consultations for GE in Norwegian primary care in the period 2006–15. The proportion of GE consultations with antibiotic treatment increased from 1.4% in 2006 to 2.2% in 2012 and then decreased to 1.8% in 2015. Fluoroquinolones (28.9%) and metronidazole (26.8%) were most frequently used. Whereas the number of fluoroquinolones courses decreased after 2012, the number of metronidazole courses continued to increase until year 2015. The antibiotic treatment proportion of GE consultations was lowest in young children and increased with increasing age.

**Conclusion:**

Antibiotic treatment is infrequently used in GE consultations in Norwegian primary care. Although there was an overall increase in use during the study period, we observed a reduction in overall use after year 2012. Young children were treated with antibiotics in GE consultations less frequent than older patients.

**Key Messages**

• Antibiotics for gastroenteritis are infrequently used in Norwegian primary care.
• Antibiotics usage increased from 2006 to 2012, followed by decrease through 2015.
• Metronidazole and fluoroquinolones were most frequently used.
• Children were least frequently treated with antibiotics for gastroenteritis.

## Introduction

Gastroenteritis (GE) is a common disease worldwide. In high-income countries, most episodes of GE are self-limiting without need of medical attention (1–4). Those seeking health care services are generally managed in primary care, accounting for about 130 000 consultations (0.9% of all primary care consultations) annually in Norway (5).

Studies from Northern European countries have shown that in primary care the infective agents are most commonly either viruses or cannot be identified (6–9). Norwegian guidelines and international recommendations state that antibiotics should be avoided for the treatment of GE in primary care (10,11). For most causal microbes, antibiotics are not shown to shorten the symptomatic phase of GE and, in some cases, could contribute to a more serious outcome (12,13). However, specific antibiotic treatment is recommended for certain gastrointestinal infections, especially in the hospital setting (11,14). In 2015, the Norwegian Government launched the Action Plan to Fight Antimicrobial Resistance in the Health Care Services (15), with the target of reducing total sales of antibiotics in human medicine by 30% within the year 2020 when compared with the level in 2012 (16). By 2015, an 11% reduction was already observed (17).

In high-income countries, GE is seldom treated with antibiotics in primary care, with prescribing proportions ranging from 5% to 11% varying between countries (18–22). In the current study, we use complete national registry data with the aim to investigate time trends and patient characteristics associated with antibiotic treatment for GE in Norwegian primary care from 2006 to 2015.

## Materials and methods

### Primary care in Norway

All residents in Norway are entitled to be on the patient list of a GP, and 99% of the population was registered to this service in 2015 (23). Most consultations in primary care, including daytime emergency consultations, are carried out in general practice surgeries during regular opening hours. In addition, emergency medical services are organized as out-of-hour (OOH) services with GPs on duty in the municipalities or as 24-hour emergency services in larger cities. In the management of infectious diseases, point-of-care C-reactive protein (CRP) testing is widely used in general practice and OOH services in Norway (24). GPs play a key role in certifying all sorts of sickness absence. Most employees will need documentation from a physician for sick leave exceeding three days. For infection control reasons, it is advised to issue sickness certificates to GE patients in specific work situations independent of the clinical manifestation and possible loss of function (food production and preparation, patient contact) (25).

We linked data from two national registries for the 10-year period 2006–15: Reimbursement claims data from Norwegian primary care (the KUHR database) and the Norwegian prescription database (NorPD).

### The KUHR database

Reimbursement claims data from both daytime general practice and OOH services are registered in the national KUHR database. The reimbursement claims include information about service type (general practice or OOH service), patient (unique personal identifier defining age and sex) and time for the consultation and diagnoses (International Classification for Primary Care [ICPC-2] codes) for each contact. Reimbursed procedures, such as point-of-care CRP testing and issuing of sickness certificates, are also included in these data, whereas no specific codes exist for microbiological testing of stool samples.

In this study, we used data from all consultations by attendance in general practice and OOH services. Home visits, and consultations made electronically or by telephone, were not included in the data set extracted from KUHR. For administrative reasons, daytime activity data from the 24-hour emergency services in Bergen (the second largest city in the country with 5% of the total population) are not registered in the KUHR database, and thus not part of this study.

We defined a gastroenteritis consultation (‘GE consultation’) as a consultation with one or more of the following ICPC-2 codes: ‘D11 Diarrhoea’, ‘D70 Gastrointestinal infection‘ and ‘D73 Gastroenteritis, presumed infection’. ‘D70 Gastrointestinal infection’ represent the most detailed level of diagnostic codes for gastrointestinal infections. We categorized patient age in the KUHR database into the following 10 categories: 0–4, 5–14, 15–24, 25–34, 35–44, 45–54, 55–64, 65–74, 75–84 and ≥85 years.

### The Norwegian Prescription Database

The NorPD is a complete registry of all prescription drugs dispensed from pharmacies in Norway. Drugs used for treatment of inpatients in hospitals and nursing homes are not registered in NorPD. NorPD contains information about the patient (pseudonym unique personal identifier), time for dispensing and information about the drug [Anatomical Therapeutic Chemical (ATC) classification system code]. We used data from the NorPD for all prescribed systemic antibiotic courses dispensed from pharmacies in Norway during the 10-year period, 2006–15.

We defined ‘course of antibiotics’ as a course of a prescribed systemic antimicrobial drug dispensed from a pharmacy and registered in the NorPD with the following ATC codes: ‘J01 Antibacterials for systemic use’, ‘A07AA09 Vancomycin’ or ‘P01AB01 Metronidazole’. We categorized antibiotics as either ‘GE relevant’ or ‘not GE relevant’, as we found it necessary to make this divide to further interpret the data. According to Norwegian and international guidelines, we defined the following antibiotics as relevant for treatment of gastrointestinal infections (‘GE relevant’): fluoroquinolones, metronidazole, macrolides, tetracycline, trimethoprim-sulphamethoxazole and vancomycin. All other antibiotics were defined as ‘not GE relevant’. When a GE consultation is linked to ‘not GE relevant’ antibiotics this can result from both inappropriate prescribing and misclassification in our data set (for example prescribing made for other diseases than GE). Additionally, we defined the following as urinary tract infection antibiotics (‘UTI antibiotics’), as their only indication is UTI: pivmecillinam, mecillinam, trimethoprim, nitrofurantoin and metenamin.

### Linking of data sets

The consultation data from the KUHR database were linked to the drug prescription data from NorPD by the patients’ pseudonym unique personal identifiers.

Due to privacy concerns, the Norwegian Data Protection Authority would not accept original dates coupled with patient data. These were therefore replaced by Statistics Norway with a random reference date unique for each patient, from which the time of each registration in this dataset refers to.

**Figure 1.**
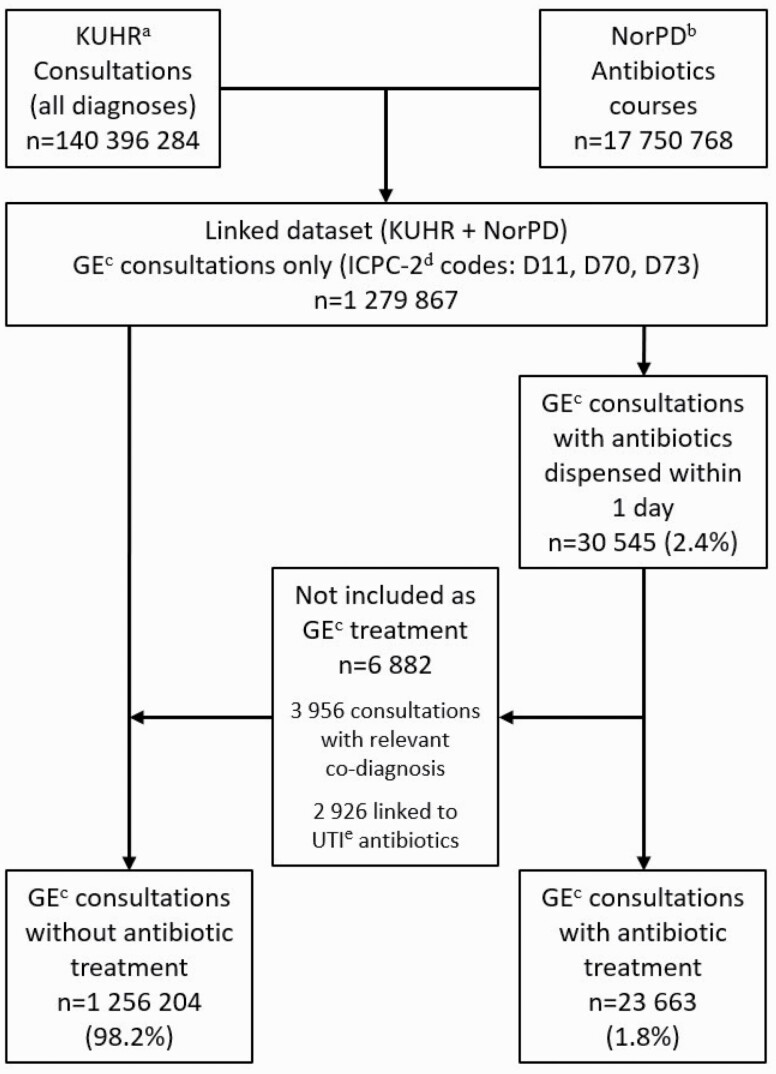
Flow chart of gastroenteritis consultations with and without antibiotic treatment in primary care. Norway, 2006–15. ^a^KUHR: Reimbursement claims database (the KUHR database). ^b^NorPD: The Norwegian Prescription Database. ^c^GE: gastroenteritis. ^d^ICPC-2: International Classification for Primary Care, version 2. ^e^UTI: urinary tract infection.

A course of antibiotics was considered as linked to a consultation in primary care when the prescribed drug was dispensed from the pharmacy at the same day or the day after the consultation. We extracted all GE consultations, and the courses of antibiotics linked to these consultations, for analyses. Both antibiotics defined as ‘GE relevant’ and ‘not GE relevant’ were included as treatment for GE in the analyses, except for the following two categories: (i) Courses of antibiotics (both ‘GE relevant’ and ‘not GE relevant’) linked to consultations with a co-diagnosis (other than D11, D70 or D73) likely to explain the prescription (Supplementary Table S1) and (ii) courses of ‘UTI antibiotics’. These courses were excluded as treatment for GE, and consultations linked to these were included as GE consultations without antibiotic treatment in the analyses (Fig. 1).

### Statistics

We calculated the proportion of GE consultations that were followed by antibiotic treatment. Patient characteristics, use of CRP and issuing of sickness certificates were compared between GE consultations with and without antibiotic treatment and between GE consultations in general practice and in OOH services. We explored time trends in the use of different antibiotics as treatment for GE. The data were analysed using StataSE 16.1 and Microsoft Excel for Windows 365 MSO.

## Results

There were 1 279 867 GE consultations in Norway in the period 2006–15, of which 84.5% (*n* = 1 081 162) were in general practice and the rest in OOH services.

Antibiotic treatment was linked to 1.8% (*n* = 23 663) of the GE consultations (Table 1), after excluding the following as GE consultations with antibiotic treatment not for GE: 3956 consultations with a co-diagnosis more relevant to the prescription (of these, 2076 were an R-diagnosis in ICPC-2, indicating a respiratory tract infection), and 2926 consultations linked to courses of UTI antibiotics (Fig. 1). In general practice, the proportion of GE consultations with antibiotic treatment was 1.8% (*n* = 19 617), and in the OOH services, the proportion was 2.0% (*n* = 4046).

**Table 1.** Characteristics of gastroenteritis consultations without and with antibiotic treatment in primary care (daytime general practice and out-of-hour services) in Norway 2006–15 (*N* = 1 279 867)

	Without antibiotic treatment						With antibiotic treatment					
	DGP +OOH		DGP		OOH		DGP + OOH		DGP		OOH	
	*n*	%	*n*	%	*n*	%	*n*	%	*n*	%	*n*	%
Total	1 256 204	100	1 061 545	84.5^a^	194 659	15.5^a^	23 663	100	19 617	82.9^a^	4046	17.1^a^
Age (years)												
Mean age	31.8		33.5		22.6		40.2		41.4		34.6	
0–4	269 130	21.4	194 917	18.4	74 213	38.1	2679	11.3	2034	10.4	645	16.0
5–14	98 085	7.8	75 769	7.1	22 316	11.5	1061	4.5	824	4.2	236	5.8
15–24	156 270	12.4	132 689	12.5	23 581	12.1	2672	11.3	2068	10.5	604	14.9
25–34	206 649	16.5	182 321	17.2	24 328	12.5	3490	14.8	2820	14.4	669	16.6
35–44	154 164	12.3	139 375	13.1	14 789	7.6	3340	14.1	2800	14.3	539	13.3
45–54	118 360	9.4	108 258	10.2	10 102	5.2	3203	13.5	2739	14.0	463	11.5
55–64	105 307	8.4	96 810	9.1	8497	4.4	3223	13.6	2812	14.3	410	10.1
65–74	72 132	5.7	65 481	6.2	6651	3.4	2217	9.4	1959	10.0	255	6.3
75–84	53 231	4.2	46 926	4.4	6305	3.2	1281	5.4	1120	5.7	158	3.9
85–	22 874	1.8	189 98	1.8	3876	2.0	497	2.1	428	2.2	67	1.6
Missing	2	0	1	0	1	0	0	0	0	0	0	0
Sex												
Male	592 800	47.2	496 546	46.8	96 254	49.5	11 361	48.0	9319	47.5	2042	50.5
Female	663 402	52.8	564 998	53.2	98 404	50.6	12 302	52.0	10 298	52.5	2004	49.5
Missing	2	0	1	0	1	0	0	0	0	0	0	0
CRP												
No	808 174	64.3	724 745	68.3	83 429	42.9	9927	42.0	8809	44.9	1118	27.6
Yes	448 030	35.7	336 800	31.7	111 230	57.1	13 736	58.1	10 808	55.1	2928	72.4
Sickness certificate^b^												
No	402 727	56.1	345 790	53.8	56 937	74.9	10 941	69.1	8820	66.5	2121	82.0
Yes	315 560	43.9	296 477	46.2	19 083	25.1	4905	31.0	4438	33.5	467	18.0
Total	718 287	100	642 267	100	76 020	100	15 846	100	13 258	100	2588	100

Distribution within sex, age, point-of-care CRP testing and sickness certificates is given by column. DGP, daytime general practice; OOH, out-of-hour services.

^a^Distribution of service types (daytime general practice and OOH services) within consultations without antibiotic treatment and with antibiotic treatment, respectively.

^b^Analyses of sickness certificates are restricted to patients aged 20–67 years.

**Figure 2.**
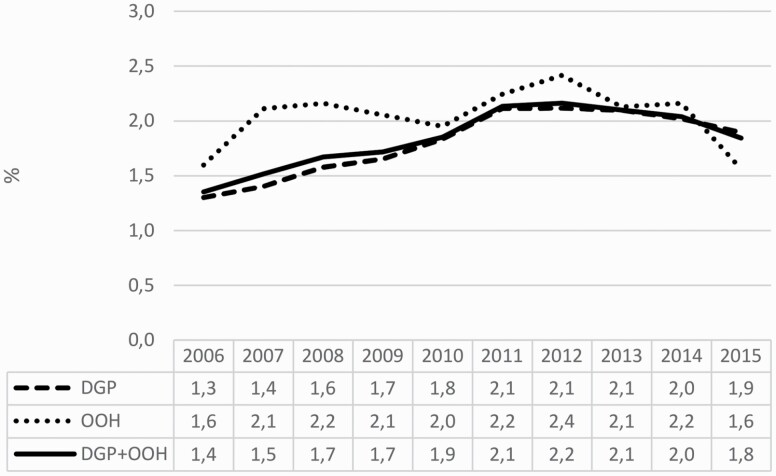
Proportion of gastroenteritis consultations with antibiotic treatment by year and type of service, Norway 2006–15. *N* = 1 279 867. DGP, daytime general practice; OOH, out-of-hour services.

The number of GE consultations with antibiotic treatment increased by 78.4% from 1636 in 2006 to 2918 in 2012, followed by a 16% decrease from 2012 until 2015. A similar pattern was observed for the proportion of GE consultations with antibiotic treatment, which increased from 1.4% in 2006 to 2.2% in 2012 and then decreased to 1.8% in 2015 (Fig. 2).

**Figure 3.**
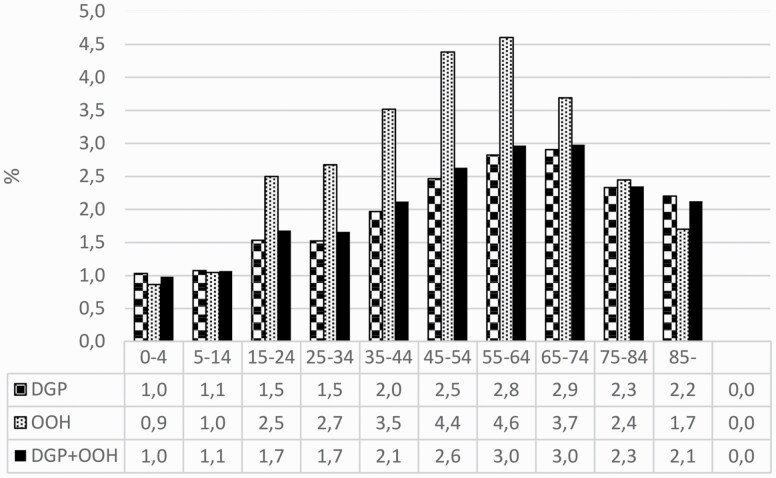
Proportion of gastroenteritis consultations with antibiotic treatment by age category and type of service, Norway 2006–15. *N* = 1 279 867. DGP, daytime general practice; OOH, out-of-hours services.

There was no difference between the sexes in proportions of GE consultations with antibiotic treatment (data not shown). The proportion of GE consultations with antibiotic treatment was lowest in patients aged 0–4 years (1.0%) and increased with increasing age up to the categories 55–64 and 65–74 years (3.0%). This trend was even more pronounced in the OOH services (Fig. 3).

CRP testing was used in 58.1% of the GE consultations with antibiotic treatment, when compared with 35.7% without antibiotic treatment (Table 1). CRP testing was used more frequently in OOH services than in general practice, this applied to both GE consultations with antibiotic treatment and without (Table 1). The proportion of CRP testing in GE consultations with antibiotic treatment increased from 52.4% in 2006 to 60.8 % in 2012 but remained stable for the years 2012–15 (data not shown).

Most of the GE consultations with antibiotic treatment were linked to single courses of antibiotics (90.3%, *n* = 21 378). A combination of two or three antibiotic courses was given following 9.6% (*n* = 2277) and 0.03% (*n* = 8) of GE consultations with antibiotic treatment, respectively. Thus, the 23 663 GE consultations with antibiotic treatment were linked to 25 956 antibiotic courses. Of these, the most frequently used ‘GE-relevant’ antibiotics were fluoroquinolones (28.9%), metronidazole (26.8%) and macrolides (10.4%). β-Lactamase-sensitive penicillins, defined as ‘not GE relevant’ antibiotics in this study, were third most frequent, accounting for 10.8%.

For the ‘GE-relevant’ antibiotics, we found an increase from 2006 to 12 in the number of courses of fluoroquinolones (128% increase), metronidazole (92.1% increase), sulfamethoxazole/trimethoprim (68.6% increase), tetracyclines (50.7% increase) and macrolides (64% increase), although there was a decrease in the number of courses of all these antibiotics from year 2012 to 2015, except for metronidazole (Table 2). For the ‘not GE-relevant’ antibiotics, an increase in the number of courses of penicillins with extended spectrum (70.8% increase) and β-lactamase-sensitive penicillins (40% increase) was found for GE consultations during the 10-year period (Table 2).

**Table 2.** Types of antibiotic courses for gastroenteritis treatment by year, Norway 2006–15 (*N* = 25 956)

	2006	2007	2008	2009	2010	2011	2012	2013	2014	2015
Fluoroquinolones	447	480	677	685	753	866	1019	976	886	701
Metronidazole	418	488	633	599	694	736	803	845	895	845
β-Lactamase-sensitive penicillins	225	201	311	222	328	323	312	296	281	315
Macrolides	222	218	240	197	284	325	364	289	317	241
Penicillins with extended spectrum	144	138	200	170	207	222	248	243	288	246
Tetracyclines	140	174	176	160	155	203	211	206	165	167
Trimethoprim-sulphamethoxazole	86	107	127	115	131	139	145	121	136	109
Other^a^	24	23	34	37	45	45	45	50	71	43
β-Lactamase-resistant penicillins	18	12	20	32	33	33	41	28	50	21
Cephalosporines	23	18	29	26	30	21	18	20	16	14
Total	1747	1859	2447	2243	2660	2913	3206	3074	3105	2702

List by total number of courses during the period.

^a^‘Other’ include vancomycin, clindamycin, fidaxomicin and fusidic acid.

Metronidazole and fluoroquinolones (38.1%, *n* = 868) represented the most frequent combination among the 2277 double courses, followed by metronidazole and extended spectrum penicillins (27.3%, *n* = 621), metronidazole and tetracyclines (15.8%, *n* = 359), and metronidazole and macrolides (9.5%, *n* = 116).

## Discussion

### Summary

We found that 1.8% of the GE consultations in Norwegian primary care resulted in treatment with antibiotics during the years 2006–15. Young children were treated with antibiotics less frequent than older patients. Fluoroquinolones and metronidazole were most frequently used, followed by β-lactamase-sensitive penicillins and macrolides. The proportion of GE consultations resulting in antibiotic treatment increased until 2012, after which it declined. The same trend with initial increase and later reduction in treatment was not seen for metronidazole.

### Strengths and limitations

The main strength of this study was the use of linked complete registry data from nearly all consultations in general practice and OOH services, and all courses of systemic antibiotics dispensed from pharmacies in Norway during a 10-year period. A limitation is that a part of the reimbursement claims from the 24-hour emergency services in Bergen (daytime consultations from workdays) are not included, leading to a minor underreporting of consultations in the OOH services. Furthermore, claims from electronic/telephone consultations or home visits were not included in the current study. We expect that the use of telephone consultations is considerable, due to the nature of GE as a contagious disease. But these are probably dominated by requests for sick leave or similar administrative purposes, and also more prone to misclassification of disease on reimbursement claims (26). However, telephone contacts may be used in the follow up of patients, and if these contacts result in the prescription of antibiotics, these courses would be missing in the current study. On the other hand, this may lead to an even greater underreporting of consultations without treatment. Hence, we do not think the study is subject to underestimation of antibiotic treatment in Norwegian primary care.

Possible misclassification of the disease (GE) may challenge the internal validity. Our definition of a GE consultation including ‘D11 Diarrhoea’ but not ‘D10 Vomiting’ is in line with the definition used by the Norwegian Syndromic Surveillance System (27), and a recent Dutch study on antibiotic treatment of GE in primary care (21). As a result, consultations for diarrhoea of other causes than GE are included, whereas GE consultations coded with ‘D10 Vomiting’ are missed. To our knowledge, studies on the validity of the diagnostic algorithm are lacking. Our calculation of treatment proportion was based on GE consultations, not GE cases or GE events. This implies that each case could have had several consultations during one GE event, leading to the possibility of an underestimation of the treatment proportion.

The data on antibiotics were based on courses dispensed from pharmacies, not prescriptions. The indirect linking of dispensing to consultations may lead to possible misclassification of antibiotics as treatment for GE. We sought to minimize this by excluding courses linked to consultations with co-diagnoses more likely to represent the real indication for the prescription, as treatment for GE. We also excluded courses of UTI antibiotics as treatment for GE for the same reason. Still, we believe that our study will include dispensing of courses misclassified as GE treatment. This could be because relevant co-diagnoses were not registered in the consultation or the course might have been prescribed in consultations not included in the data material, such as telephone consultations, home visits, consultations with doctors outside primary care, or in consultations taking place between the GE consultation and the dispensation. Antibiotic courses may also have been incorrectly defined as treatment for GE if the consultation was misclassified as a GE consultation.

### Interpretation of results

The antibiotic treatment proportion in our study was lower (1.8%) than presented in literature from other high-income countries (18–22). This can be explained by low levels of bacterial and parasitic gastrointestinal infections in Norway, relative to viral infections (28,29). Other possible explanations can be that Norway generally has a low consumption of antibiotics (30), different health care seeking behaviour, or that GE cases with high risk of severe illness are hospitalized and thus not included in the study. The observed declining trend in antibiotic use in GE consultations after 2012 (16% reduction) coincides with an observed reduction in the total use of antibiotics (11% reduction) in Norway during the same period (17) and is in accordance with the goals of the Norwegian Action Plan (15).

Due to lack of clinical and microbiological data, we do not know the real indications for the antibiotic courses, and even less whether the treatment was empirical or specific. Our finding of relatively infrequent use of antibiotics in GE consultations indicates a restrictive use of antibiotics in the treatment of GE, as recommended by guidelines. The most frequently used antibiotics in the GE consultations in our study were fluoroquinolones and metronidazole, which are antibiotics shown to be commonly prescribed for gastrointestinal infections in studies from primary care in the Netherlands, Switzerland and England (21,22,31). We have no explanations for the continuous increase in the use of metronidazole after 2012.

We found a lower prescription proportion among the youngest patients, a finding in line with a recent study from the Netherlands (21). This may be explained by higher GE consultation frequency, and the increased likelihood of viral aetiology in younger patients (5).

The frequent use of the ‘not GE relevant’ β-lactamase-sensitive penicillins may be surprising as they are not suitable for treatment of any gastrointestinal infections, although they are strongly advocated as the antibiotics of choice in treatment for several other infections commonly seen in primary care. A study from the UK found β-lactamase-sensitive penicillins account for 1.3% of antibiotic prescriptions for infections in the gastrointestinal tract, while a Dutch study of antibiotic treatment for GE in primary care did not include prescriptions of β-lactamase-sensitive penicillins (21). A proportion of the use of ‘not GE relevant’ antibiotics is probably related to misclassification of disease and/or antibiotic treatment for GE. Fifty percent of the treatments with β-lactamase-sensitive penicillins in the present study were linked to patients under 15 years of age. This may reflect a greater diagnostic challenge in consultations with children, with high levels of co-infections and uncertain symptoms and findings, leading to more misclassification of disease in these age categories. However, we cannot rule out the possibility that some doctors inappropriately prescribed the drug as a first-line drug with the intention to treat GE.

Previous studies from other European countries have indicated higher prescription rates in OOH services than in general practice for several infections (32–34), which corresponds to our finding of higher antibiotic treatment proportion in GE consultations in the OOH services.

The extensive use of CRP testing in Norwegian primary care, especially in consultations with patients with suspected infection and in OOH services, is described in previous studies from Norway (24,35,36). We do not have clinical information about the reason for our finding of extensive use of CRP testing in GE consultations with antibiotic treatment, nor the results of the tests, or if the tests affected the decision whether to prescribe antibiotics.

## Conclusions

Antibiotic treatment is used in a very small proportion of GE consultations in Norwegian general practice and OOH services. Although there was an overall increase in use during the study period, there was a reduction in overall use after year 2012. There was a reduction in use of fluoroquinolones and macrolides, but an increase in metronidazole used also after 2012. The antibiotic treatment proportion of GE consultations was lowest in young children and increased with increasing age.

## Supplementary Material

cmab080_suppl_Supplementary_Table_S1

## Data Availability

The data underlying this article cannot be shared publicly due to limitations given by the ethical approval and the data license granted by the Regional Committee for Medical and Health Research Ethics and the Norwegian Data Protection Agency, respectively.

## Declaration

Funding: Faculty of Medicine at the University of Bergen; Research Unit for General Practice at NORCE Norwegian Research Centre.

Ethical approval: Regional Committee for Medical and Health Research Ethics, REC West (project number 2016/559); the Norwegian Data Protection Agency (project number 16/01083).
