# Supplementary material for: Antibiotics for gastroenteritis in general practice and out-of-hour services in Norway 2006–15
Source: Fam Pract. 2021 Jul 15;39(1):19–25. doi: 10.1093/fampra/cmab080 (PMC8769278; doi:10.1093/fampra/cmab080)
Supplement: cmab080_suppl_Supplementary_Table_S1 [file cmab080_suppl_supplementary_table_s1.docx]

**TABLE S1 SUPPLEMENTARY.** International Classification for Primary Care, version 2 (ICPC-2) co-diagnoses (other than D11, D70 and D73) more likely representing the indication for prescription of systemic antibiotics in primary care. List by order of appearance in ICPC-2.

| **General and unspecified** |
| --- |
| A78 Infectious disease other |
| **Eye** |
| F72 Blepharitis |
| F73 Eye infection/inflammation other |
| **Ear** |
| H01 Ear pain |
| H29 Ear symptom/complaint other |
| H70 Otitis externa |
| H71 Acute otitis media |
| H72 Serous otitis media |
| H73 Eustachian salpingitis |
| H74 Chronic otitis media |
| **Cardiovascular** |
| K70 Infection of circulatory system |
| K71 Rheumatic fever |
| **Musculoskeletal** |
| L70 Infections musculoskeletal system |
| **Neurological** |
| N71 Meningitis/encephalitis |
| N73 Neurological infection other |
| **Respiratory** |
| R05 Cough |
| R09 Sinus symptom/complaint |
| R21 Throat symptom/complaint |
| R25 Sputum/phlegm abnormal |
| R71 Whooping cough |
| R72 Strep throat |
| R73 Boil/ascess nose |
| R74 Upper respiratory infection acute |
| R75 Sinusitis acute/chronic |
| R76 Tonsillitis acute |
| R77 Laryngitis/tracheitis |
| R78 Acute bronchitis/bronchiolitis |
| R79 Chronic bronchitis |
| R81 Pneumonia |
| R82 Pleurisy/pleural effusion |
| R83 Respiratory infection other |
| **Skin** |
| S09 Infected finger/toe |
| S10 Boil/carbuncle |
| S11 Skin infection post-traumatic |
| S13 Animal/human bite |
| S73 Pediculosis/skin infestation other |
| S76 Skin infection other |
| S84 Impetigo |
| S94 Ingrowing nail |
| S96 Acne |
| **Urological** |
| U01 Dysuria/painful urination |
| U07 Urine symptom/complaint other |
| U13 Bladder symptom/complaint other |
| U29 Urinary symptom/complaint other |
| U70 Pyelonephritis |
| U71 Cystitis |
| U72 Urethritis |
| U99 Urinary disease, other |
| **Pregnancy, Childbearing, Family Planning** |
| W29 Pregnancy symptom/complaint other |
| W70 Puerperal infection/sepsis |
| W71 Infection complicating pregnancy |
| W94 Puerperal mastitis |
| **Female Genital** |
| X14 Vaginal discharge |
| X15 Vaginal symptom/complaint other |
| X17 Pelvis symptom/complaint female |
| X23 Fear of sexually transmitted disease (f) |
| X29 Genital symptom/complaint female other |
| X70 Syphilis female |
| X71 Gonorrhoea female |
| X73 Genital trichomoniasis female |
| X74 Pelvic inflammatory disease |
| X92 Chlamydia infection genital female |
| X99 Genital disease female, other |
| **Male Genital** |
| Y02 Pain in testis/scrotum |
| Y03 Urethral discharge |
| Y04 Penis symptom/complaint other |
| Y05 Scrotum/testis symptom/complaint other |
| Y06 Prostate symptom/complaint |
| Y25 Fear of sexually transmitted disease male |
| Y29 Genital symptom/complaint male other |
| Y70 Syphilis male |
| Y71 Gonorrhoea male |
| Y73 Prostatitis/seminal vesiculitis |
| Y74 Orchitis/epididymitis |
| Y75 Balanitis |
| Y99 Genital disease male, other |
